# Cryptococcosis by *Cryptococcus neoformans/Cryptococcus gattii S*pecies Complexes in non-HIV-Infected Patients in Southeastern Brazil

**DOI:** 10.1590/0037-8682-0169-2021

**Published:** 2021-09-06

**Authors:** Erika Nascimento, Patrícia Helena Grizante Barião, Marcia Regina von Zeska Kress, Fernando Crivelenti Vilar, Rodrigo de Carvalho Santana, Gilberto Gambero Gaspar, Roberto Martinez

**Affiliations:** 1 Universidade de São Paulo, Faculdade de Medicina de Ribeirão Preto, Departamento de Clínica Médica, Ribeirão Preto, SP, Brasil.; 2 Universidade de São Paulo, Faculdade de Ciências Farmacêuticas de Ribeirão Preto, Departamento de Análises Clínicas, Toxicológicas e Bromatológicas, Ribeirão Preto, SP, Brasil.

**Keywords:** Cryptococcal disease, Cryptococcal meningitis, *Cryptococcus neoformans* complex, *Cryptococcus gattii* complex

## Abstract

**INTRODUCTION:**

The clinical manifestations of cryptococcosis are usually associated with the infecting agents *Cryptococcus neoformans* (CN) and *C. gattii* (CG) species complexes and the host. In this study, non-HIV-infected patients, at a university hospital in southeastern Brazil, had epidemiological and clinical data associated with cryptococcal disease and isolated *Cryptococcus* species: CN - 24 patients and CG - 12 patients.

**METHODS:**

The comparison was comprised of demographic data, predisposing factors, clinical and laboratory manifestations, and outcomes of cryptococcosis patients treated between 2000 and 2016. Immunocompetent and immunosuppressed patients were also compared, irrespective of the infecting species. *Cryptococcus* spp. were genotyped by PCR-RFLP analysis of the *URA5* gene.

**RESULTS:**

Infections by the CN species complex (100% VNI genotype) were associated with drug immunosuppression and fungemia, and patients infected with the CG species complex (83% VG II and 17% VGI genotypes) had more evident environmental exposure and higher humoral response. CN and CG affected patients with or without comorbidities.

**CONCLUSIONS:**

Diabetes mellitus, other chronic non-infectious diseases, and alcoholism were likely predisposing factors for infection by both CN and CG species. Immunocompetent patients, independent of the infecting *Cryptococcus* species complexes, showed a higher occurrence of meningitis and a trend toward less fungal dissemination and longer survival than immunosuppressed hosts.

## INTRODUCTION

In the last two decades, there has been a decline in the occurrence of opportunistic cryptococcosis in AIDS cases with a simultaneous increase in the incidence of cryptococcal disease in non-HIV-infected patients^1^ ^,^ ^2^. HIV seronegative patients infected with *Cryptococcus* spp. are a heterogeneous population that includes cases of therapeutic immunosuppression, comorbidities, solid organ transplantation, and immunocompetent individuals with no apparent comorbidity. Differences in the clinical characteristics and lethality of cryptococcal disease have been observed between these groups of patients and with cryptococcosis associated with AIDS^3^ ^,^ ^4^.

Cryptococcal disease is caused predominantly by species of *C. neoformans* (CN) and *C. gattii* (CG) complexes. The CN complex includes the species *C. neoformans* (genotype VNI/VNII/VNB), *C. deneoformans* (genotype VNIV), and a hybrid species (genotype VNIII). CG complex includes *C. gattii strictu senso*, *C. deuterogattii*, *C. bacillisporus*, *C. tetragattii*, and *C. decagattii*, respectively, genotypes VGI, VGII, VGIII, VGIV, and VGV/IIIc; hydrids between species of the two *Cryptococcus* complexes have been reported^5^. CN is more prevalent and has a wider geographical distribution, while CG is more isolated in tropical and subtropical regions, although species of this complex have also been isolated in temperate climate environments^6^. CG is often associated with infections in immunocompetent individuals, in addition to more frequent lung and brain parenchyma lesions^7^ ^,^ ^8^. Different geographical areas may show differences in the predominant genotype/species of *Cryptococcus* spp. and eventually in the clinical presentation of cryptococcal disease^9^. *C. neoformans* molecular type VNI is the major agent of cryptococcal disease in Brazil, followed by *C. gattii*, and the prevalence of this last species increases from the southern to northern region of the country^6^.

This study aimed to assess the characteristics of cryptococcosis in non-HIV-infected patients in southeastern Brazil. The clinical aspects of cryptococcal disease in immunocompetent individuals and the comparison of cases with isolation of CN and CG species complexes were analyzed. This study has clinical relevance because of the scarcity of studies on cryptococcosis comparing CN or CG complex infections in non-HIV-infected patients, including immunocompetent individuals, from Brazil.

## METHODS

This retrospective study analyzed the clinical and epidemiological data of non-HIV-infected patients with cryptococcal disease. The patients received medical assistance between 2000 and 2016 at the University Hospital of the Ribeirão Preto Medical School, University of São Paulo (SP), and lived in the region of Ribeirão Preto, SP, Brazil. The data were analyzed according to the results of the *Cryptococcus* spp. genotyping, which were divided into two groups: 1) the CN group with 24 patients infected with species of the *C. neoformans* complex; and 2) the CG group with 12 patients infected with species of the C. *gattii* complex. Further analysis compared data from immunocompetent (apparently healthy) patients (10/36) with other patients with comorbidities and/or immunosuppressed patients (26/36). Three other patients were excluded due to a lack of clinical data, or because the isolation of *Cryptococcus* spp. was considered as only colonization. 

Clinical and epidemiological data were collected from patients’ medical records, including age, sex, underlying diseases, and predisposing factors for cryptococcal disease. The involvement of organs and tissues by *Cryptococcus* spp. was assessed by clinical manifestations, radiographic images, the isolation site of this yeast, cerebrospinal fluid (CSF) analysis, and biopsy of the lung, skin, and lymph nodes. Antifungal treatment for meningitis and bloodstream infection was performed with deoxycholate amphotericin B (CN = 11/17; CG = 4/7) or liposomal amphotericin B (CN = 6/17; CG = 3/7) and was maintained until there was no fungal growth in the CSF, and these drugs were associated or not with fluconazole. The consolidation and maintenance phases of the antifungal therapy were performed using fluconazole. Patients with lung and skin lesions and without meningitis were treated orally with fluconazole or itraconazole. The outcome was determined one year after diagnosis, and cases were classified as Cure-Improvement or Death.

*Cryptococcus* spp. were isolated from the following clinical samples: CSF (n=23), blood (n=9), skin biopsy (n=4), bronchoalveolar lavage (n=2), and a sample of each of the following materials: sputum, lung biopsy, pleural fluid, lymph node biopsy, and urine. Sabouraud dextrose agar with or without chloramphenicol was used to isolate the fungus, and Bact Alert (Biomérieux Brasil) or BD (Becton Dickinson and Company, USA) flasks were used for blood culture. Identification of the genus *Cryptococcus* was carried out by conventional laboratory methods of clinical mycology and/or the automated Vitek (BioMérieux Brasil) system. Clinical isolates of *Cryptococcus* spp. were maintained in the laboratory using periodic subcultures.

Molecular identification of CN/CG species complexes was carried out by polymerase chain reaction (PCR) using pairs of specific primers that amplify DNA fragments to 695 bp for *C. neoformans* (CNa-70a/CNa-70s) and 448 bp for *C. gattii* (CNb-49a/CNb-49s)^10^. The molecular types of the CN (VNI, VNII, VNIII, and VNIV) and CG complexes (VGI, VGII, VGIII, and VGIV) were assessed by restriction fragment length polymorphism (RFLP) of the *URA5* gene. After amplification, the products were subjected to enzymatic restriction with the restriction endonucleases *Hha*I (Invitrogen, Thermo Fisher) and *Cfr*13I (Invitrogen,Thermo Fisher)^11^ ^,^ ^12^. PCR-RFLP patterns were assigned visually by comparing them to the standard strains *C. neoformans*, molecular type VNI (WM148); *C. neoformans*, molecular type VNII (WM626); *C. neoformans* × *C. deneoformans* hybrid, molecular type VNIII (WM628); *C. deneoformans*, molecular type VNIV (WM629); *C. gattii*, molecular type VGI (WM179); *C. deuterogattii*, molecular type VGII (WM178); *C. bacillisporus*, molecular type VGIII (WM175), and *C. tetragattii*, molecular type VGIV (WM779) from the Laboratory of Mycology (Pathogenic Fungi Collection) at the Oswaldo Cruz Foundation (FIOCRUZ)-INI/FIOCRUZ in Brazil.

The titer of the cryptococcal antigen in the CSF of 18 patients was determined by the latex agglutination method using the CALAS^®^ Kit (Meridian Bioscience, USA). The titer of anti-*Cryptococcus* antibodies in the serum of 27 patients was measured by counterimmunoelectrophoresis using in-house prepared antigen, which were obtained by sonicating four samples of clinical isolates of *C. neoformans* (identified by cultivation in L-canavanine-glycine-bromothimol blue medium).

Statistical analysis was performed using GraphPad Prism v.6 (GraphPad Software, La Jolla, CA, USA). The proportions were compared using the chi-square test or Fisher’s exact test. The Mann-Whitney U test was used to assess CSF parameters and the titer of the serum anti-*Cryptococcus* spp. antibody. The significance level was set at *P* < 0.05.

The research project was approved by the Research Ethics Committee of University Hospital, Ribeirão Preto Medical School (No. 12247/2010).

## RESULTS

Thirty-six cases of cryptococcosis in non-HIV-infected patients in southeastern Brazil were analyzed in this study. Among the patients, 72.3% (26/36) were individuals with comorbidities and/or immunosuppressed and 27.7% (10/36) were immunocompetent (apparently healthy) patients. Strains isolated from the CN group were 100% (24/24) of the VNI genotype (*C. neoformans*). The CG group consisted of 17% (2/12) of the VGI genotype (*C. gattii sensu stricto*) and 83% (10/12) of the VGII genotype (*C. deuterogattii*).

Age, sex, and associated conditions of the patients showed no significant differences between the CG and CN groups. The proportion of immunocompetent patients was higher in the CG group (Table 1). Patients from the CG group reported more prolonged exposure in a rural environment and/or working with wood or raising birds (*P*=0.0002). Immunosuppression by corticosteroids or cytotoxic drugs was observed only in the CN group, while alcoholism and diabetes mellitus were observed in patients from both groups (Table 1).

**TABLE 1:** Demographic data, associated conditions, and predisposing factors according to the *C. neoformans/C. gattii* species complex.

	C. gattii*	*C. neoformans*	Total	*P* value**
	n/%	n/%	n/%	
Age - median (range)	46.5 (4 - 73)	46.0 (2.5 - 80)	46.5 (2.5 - 80)	0.7355
Gender (Male: Female)	10:2	16:8	26:10	0.6667
Associated Conditions	7/58	19/79	26/72	0.2474
Diabetes mellitus	4/33	4/17	8/22	0.3974
Malignancy^a^	0/0	5/21	5/14	0.1494
Chronic visceral diseases^b^	6/50	11/46	18/50	1.0
Systemic erythematosus lupus	0/0	2/8	2/6	0.5429
Kidney transplantation	0/0	3/13	3/8	0.5361
Non-associated conditions	5/42	5/21	10/28	0.2474
Predisposing factors				
Environmental exposition^c^	10/83	4/17	14/39	0.0002
Pharmacologic immunosuppression	0/0	6/25	6/17	0.0793
Alcoholism	3/25	3/13	6/17	0.3781
Malnutrition	0/0	3/13	3/8	0.5361
Patients - Total	12/100	24/100	36/100	

a) Hematologic malignancies or solid organ neoplasm; b) chronic diseases and/or dysfunction in one of the following organs: lungs, liver, kidney, heart, brain, intestine; c) living or working in rural areas and/or regular contact with birds or wood; * *C. gattii* = *C. deuterogattii* (n=10), *C. gattii s.s.* (n=2); **statistical analysis.

Meningitis was the most common clinical manifestation in both of the groups. Cryptococcemia occurred only in the CN group (*P*=0.0163), while a trend towards a higher proportion of lung and skin lesions, in addition to a patient with generalized lymphadenopathy, was observed in the CG group (Table 2). Patients in the two groups presented no differences in cellularity, glucose, protein, and cryptococcal antigen levels in the CSF. Patients in the CG group showed higher serum reactivity and slightly higher antibody titers against *Cryptococcus* spp. antigens (Table 2).

**TABLE 2:** Clinical and laboratory manifestations of the cryptococcal disease according to the *C. neoformans/C. gattii* species complex.

	***C. gattii* complex**	*C. neoformans*	Total	*P* value*
	n/%	n/%	n/%	
Clinical Manifestation				
Meningitis	7/58	15/63	22/61	1.0
Brain granuloma or pseudocyst	3/25	1/4	4/11	0.0980
Cryptococcemia	0/0	9/38	9/25	0.0163
Pulmonary lesion	8/67	9/38	17/47	0.1582
Cutaneous lesion	3/25	3/13	6/17	0.3781
Lymphadenopathy	1/8	0/0	1/3	0.3333
				
CSF alterations	(n=7)	(n=15)	(n=22)	
Cells - no./µL - mean ± SD	100.42 ± 11.99	115.73 ± 236.80	110.8 ± 80.05	0.6867
Protein - mg/dL - mean ± SD	161.28 ± 236.80	144.42 ± 182.22	150± 196.1	0.8582
Glucose - mg/dL - mean ± SD	45.85± 45.38	30.28 ± 24.05	35.4 ± 32,4	0.3115
Cryptococcal antigen titer^a^ - median ange)	≥ 4096	3072	≥4096	0.4714
	(64 - ≥ 4096)	(NR - ≥ 4096)	(NR - ≥ 4096)	
Antibodies anti-*Cryptococcus* in serum^b^				
Reactive patients / total	7;11/64	4;16/25	11;27/41	0.0608
Antibodies titer - median (range)	8 (1 - 16)	2.5 (1 - 8)	4 (1 - 16)	0.0264
Patients - Total	12/100	24/100	36/100	

a) Latex agglutination test: titer-inverse of CSF dilution; b) counterimmunoelectrophoresis test: titer-inverse of serum dilution; *statistical analysis; **SD:** standard deviation; **CSF:** cerebrospinal fluid; **NR:** non-reactive.

Patients in the CN and CG groups received similar antifungal treatments, except for the use of amphotericin B monotherapy in six patients in the CN group. Lethality was higher in the CN group (48%) than in the CG group (18%), without reaching statistical significance. Cure/improvement in the CG group was verified in 2/2 patients with *C. gattii s.s.* infection and in 5/7 cases of *C. deuterogattii* infection. The period between the diagnosis of cryptococcosis and death was significantly shorter in the CN group (Table 3). Sequelae occurred in three patients: in the CG group, a child had amaurosis and delay in neuromotor development, and another patient had hypoacusis. In the CN group, amaurosis occurred in one patient.

**TABLE 3:** Antifungal drug treatment and outcome of patients with cryptococcosis due to the *C. neoformans/C. gattii* species complex.

	*C. gattii* complex	*C. neoformans*	Total	*p* value*
	n/%	n/%	n/%	
Antifungal treatment				
Amphotericin B → Fluconazole	4/33^a^	5/21	9/25	0.4428
Amphotericin B + Fluconazole	3/25	6/25	9/25	1.0
Amphotericin B	0/0	6/25	6/17	0.0793
Fluconazole	4/33	3/13	7/19	0.1904
Itraconazole	0/0	1/4	1/3	1.0
No antifungal	1/8	3/13	4/11	1.0
Outcome				
Cure/improvement^b^	9/82	12/52	21/62	0.1398
Death^b^	2/18	11/48	13/38	
Diagnosis - death time (days)				
median (range)	53 (31-75)	7 (2-79)	12 (2-79)	0.0803
Unknown	1	1	2	
Sequels	2/17	1/4	3/8	0.2527
Patients - Total	12/100	24/100	36/100	

a) One patient used ketoconazole instead of fluconazole; b) survival and lethality rates excluded patients whose outcome is unknown; * statistical analysis.

Table 4 compares cryptococcal disease in patients with the presence or absence of comorbidities or organ transplants. Immunocompetent patients (absence of comorbidity or organ transplantation) had cryptococcosis caused by both CG and CN species complexes and a higher frequency of meningitis (90%) (*P*=0.0245). Lethality was higher in patients with comorbidities (46%) than in immunocompetent patients (20%), although the difference was not statistically significant (Table 4).

**TABLE 4:** Cryptococcal disease and outcome of patients with or without comorbidities and organ transplantation, regardless of the *Cryptococcus* species.

	Comorbidity or organ transplantation		*P*-value*
	Absent n/%	Present n/%	
*Cryptococcus* species complex			
*C. gattii* complex	5/50	7/27	0.2474
*C. neoformans*	5/50	19/73	
Cryptococcosis site			
Meningitis	9/90	12/46	0.0245
Cryptococcemia	1/10	8/31	0.3921
Pulmonary	2/20	14/54	0.1326
Cutaneous	1/10	5/19	0.6546
Lymphadenopathy	0/0	1/4	1.0
Outcome^a^			
Cure/improvement	8/80	13/54	0.2508
Death	2/20	11/46	
Unknown	0	2	-
Patients - Total	10/100	26/100	

Cure/improvement and death rates excluded patients whose outcome was unknown; *statistical analysis.

## DISCUSSION

The cryptococcal disease of non-HIV-infected patients evaluated in this study was mainly related to the genotypes VNI of CN (*C. neoformans*) and VGII of CG (*C. deuterogattii*), a finding similar to that observed in clinical isolates in Brazil^6^ ^,^ ^13^. The CG species complex has been associated with immunocompetent individuals^14^, but our study revealed that *C. neoformans* also infects such people, although they are more prevalent in comorbid or organ-transplanted patients. Immunocompetent individuals who are apparently healthy may have small defects in their immune capacity, facilitating cryptococcal infection^15^. In the studied cases, 72% of the patients had previously altered health conditions, and both patients infected with the CN and CG species complex had similar rates of chronic non-infectious diseases. The immunocompetent cases (42%) and comorbidity rates in patients infected by the CG species complex were similar to those found in a large series of cases in Canada^16^. Only patients infected by *C. neoformans* had neoplasms and immunosuppression by corticosteroids or cytotoxic drugs, which suggests some specificity in the pathogenesis of the disease caused by different species of *Cryptococcus*. However, the disease caused by the CG species complex has already been associated with severe immunosuppression^17^. Diabetes mellitus and alcoholism are likely predisposing factors for the disease caused by both the CN and CG species complexes^18^ ^,^ ^19^. Exposure to environmental sources that contain *Cryptococcus* spp. was associated with *C. gattii* infection, similar to that observed in Australia^7^, in which many patients lived in rural areas. The CG group included a child with cryptococcal meningitis caused by *C. deuterogattii* (VGII genotype) after returning from a trip to a CG species complex endemic area in northeastern Brazil. *C. gattii s.s.* was isolated from a patient who caught wild birds and presented with Moyamoya disease and generalized cryptococcal lymphadenopathy. *C. gattii s.s.* was also isolated from an immunocompetent man who presented with a chronic cutaneous ulcer and had past contact with house birds.

Meningitis and disseminated disease are the most common clinical manifestations in non-HIV-infected patients, but CG species can lead to a predominance of lung involvement or cause primary skin lesions^9^ ^,^ ^20^. The frequency of meningeal, lung, and skin lesions showed no difference between the CN and CG group. A significant difference was found in bloodstream infection, which occurred only in patients infected with *C. neoformans.* This is probably a consequence of more severe immunosuppression in patients in the CN group, facilitating fungal dissemination. The frequency of patients with cryptococcal fungemia was higher in this investigation (25%) than in other Brazilian report of cryptococcosis in non-HIV-infected and non-transplanted patients (6.8%)^21^. This lower percentage is probably due to the high proportion of immunocompetent patients and CG complex infections in the later study.

The higher level of serum anti-*Cryptococcus* antibodies among patients infected by CG could be related to the lower frequency of immunosuppression in the patients in this group. A previous study found a higher humoral response of IgG and IgA antibodies in patients infected with *C. gattii* than in those infected with *C. neoformans* ^22^.

The outcome of antifungal treatment with amphotericin B and/or azole drugs showed a trend towards lower lethality among patients infected by CG species. The overall lethality in patients in this study (38%) was higher than the 21% lethality found in another series of cryptococcosis cases in non-HIV-infected patients in Brazil, although that study had a higher percentage of immunocompetent individuals^21^. The death of patients at the beginning of antifungal treatment has been observed in other hospitals and has been associated with cryptococcemia and high lactate levels in CSF^23^ ^,^ ^24^. Patients with cryptococcal disease due to CG species showed a lethality after 12 months which reached 18% in this study, 23.3% in a British Colombia-Canada study, and was more elevated in some series of cases that included immunosuppressed individuals and children^16^ ^,^ ^25^ ^,^ ^26^. 

The comparison of clinical manifestations possibly attributable to the type of causative agent, CN or CG complexes, may have been impaired by the higher proportion of immunosuppressed patients in the CN group. Thus, the clinical picture and outcome were compared between immunocompetent patients and those with previous diseases and/or immunosuppression, regardless of the *Cryptococcus* species. Cryptococcal meningitis was most commonly seen among immunocompetent patients, with a trend for cryptococcemia and pulmonary involvement manifested mainly in cases with comorbidities and/or immunosuppression. The high proportion of clinically expressed meningitis may be a consequence of the high immunological reactivity of immunocompetent patients. Tissue injury and damage in cryptococcosis are more likely to occur when the immune response of the host is very weak or very intense^27^. Immunocompetent patients also had lower lethality, although the difference was not statistically significant. HIV-infected patients with controlled or active disease (AIDS) showed similar lethality among those infected with *C. gattii* or non-*C. gattii* species^28^. Such data suggest that not only the *Cryptococcus* species but also the health condition and immunological capacity of the host are important in defining the clinical presentation and outcome of patients^14^.

This study was limited by the small number of cases, making it difficult to differentiate patient groups based on the analyzed parameters. Other cases of cryptococcosis in non-HIV-infected patients were recognized at the institution during the same period, but without the availability of isolated microorganisms for genotyping.

In conclusion, cryptococcal disease caused by *C. neoformans* (VNI genotype) was associated with immunodepressed patients and fungemia, and patients infected with *C. deuterogattii* and *C. gattii s.s.* (genotypes VGII and VGI of CG) were exposed to environmental sources of *Cryptococcus* spp. and showed a higher humoral immune response. Chronic non-infectious diseases, diabetes mellitus, and alcoholism were likely predisposing factors for infection by both CN and CG species. Immunocompetent patients (without comorbidity or solid organ transplantation) showed a high incidence of cryptococcal meningitis, a trend toward less fungal dissemination, and longer patient survival, regardless of the infecting species. The clinical expression and outcome of cryptococcal disease in non-HIV-infected patients are probably more related to the health and immunological conditions of the host than to the *Cryptococcus* species complexes.
